# ADAM10 and ADAM17—Novel Players in Retinoblastoma Carcinogenesis

**DOI:** 10.3390/ijms232012621

**Published:** 2022-10-20

**Authors:** Dario Van Meenen, Annika Doege, Emily Alefeld, André Haase, Manfred Beier, Tobias Kiefer, Eva Biewald, Klaus Metz, Oliver Dräger, Maike Anna Busch, Nicole Dünker

**Affiliations:** 1 Center for Translational Neuro- and Behavioral Sciences (C-TNBS), Institute of Anatomy II, Department of Neuroanatomy, Medical Faculty, University of Duisburg-Essen, 45147 Essen, Germany; 2 Institute of Human Genetics, Medical Faculty, Heinrich-Heine University Düsseldorf, 40225 Düsseldorf, Germany; 3 Department of Ophthalmology, Medical Faculty, University of Duisburg-Essen, 45147 Essen, Germany; 4 Institute of Pathology, Medical Faculty, University of Duisburg-Essen, 45147 Essen, Germany; 5 Institute of Cellular Neurophysiology, Medical Faculty, University of Bielefeld, 33615 Bielefeld, Germany

**Keywords:** retinoblastoma, ADAM10, ADAM17, L1CAM, CAM assay, carcinogenesis, tumorigenesis

## Abstract

A disintegrin and metalloproteinase (ADAM) family proteins, acting as sheddases, are important factors in a number of pathologies, including cancer, and have been suggested as promising therapeutic targets. The study presented focuses on the involvement of ADAM10 and ADAM17 in retinoblastoma (RB), the most common malignant intraocular childhood tumor. A significant correlation between ADAM17 expression levels and RB laterality and RB staging was observed. Levels of ADAM10 or ADAM17 regulating miRNAs miR-145, -152, and -365 were significantly downregulated in RB cell lines, and reduced miR levels with simultaneously upregulated ADAM10 and ADAM17 expression were found in RB patients. The involvement of both ADAMs analyzed in ectodomain shedding of the neuronal cell adhesion molecule L1 (L1CAM), shown to induce pro-tumorigenic effects in RB, was confirmed. Lentiviral ADAM10 and ADAM17 single or ADAM10/17 double knockdown (KD) induced caspase-dependent apoptosis and reduced cell viability, proliferation, growth, and colony formation capacity of RB cells. Moreover, differential phosphorylation of the serine/threonine kinase AKT was observed following ADAM17 KD in RB cells. Chicken chorioallantoic membrane (CAM) assays revealed that ADAM17 and ADAM10/17 depletion decreases the tumorigenic and migration potential of RB cells in vivo. Thus, ADAMs are potential novel targets for future therapeutic RB approaches.

## 1. Introduction

A disintegrin and metalloproteinase (ADAM) protein family comprises type-I transmembrane and soluble glycoproteins with several cellular functions, including adhesion, migration, proteolysis, and signaling [1,2,3]. Most ADAM metalloproteinases are membrane-anchored enzymes with a functional involvement in the ectodomain shedding of cell-surface proteins, including growth factors and cell adhesion molecules [4]. Apart from shared structural similarities, each ADAM member has different variable domains ensuring both function and tissue specificity. The domain structure and function of each ADAM protein have been described in detail by Takeda and colleagues [4]. Currently, 37 ADAMs have been identified in rats, 34 ADAMs in mice, and 22 ADAMs have been discovered in the human genome [5]. However, only 12 of the human ADAM members (ADAM8, 9, 10, 12, 15, 17, 19, 20, 21, 28, 30, and 33) contain a functional catalytic consensus sequence [4]. The function of the proteinase inactive ADAM family members is largely unknown, suggesting roles in development and as adhesion molecules instead of protease activity [6,7]. ADAMs are widely expressed in mammalian tissues, and knockout mice display variable phenotypes, whereby ADAM10, ADAM17, and ADAM19 are the only essential ADAMs in murine development [3,4].

Active ADAM proteases have multiple substrates in different tissues and organs. In this context, the C-shaped arm of ADAMs, including the metalloproteinase M-domain, the disintegrin D-domain, and the cysteine-rich C-domain, is crucial for ADAM function and substrate recognition, as well as proteolytic processing and degradation [5]. The metalloproteinase M-domain can conduct ADAMs’ cleavage function as a sheddase or proteolytic enzyme, hence processing proteolytic cleavage of extracellular domains of membrane-bound molecules. Membrane shedding provokes different effects: (I) release of the extracellular domain of a membrane-bound molecule into the body circulation, and (II) inactivation of a receptor and its corresponding pathway. In addition, ADAMs also regulate various intracellular signaling pathways [5].

ADAM10 and ADAM17, both lacking the EGF-like region found in most other ADAMs, share not only very similar sequences and crystal structures but also similarities in some of their functions [5]. ADAM17 was first described in 1997 as a TNFα converting enzyme (TACE), and subsequently, ADAMs became known as sheddases [8]. It has been shown that ADAM10 is an important player in Notch and Eph/ephrin pathways. By inducing Notch cleavage, ADAM10 triggers regulated intramembrane proteolysis (RIP), which in turn regulates gene transcription and mRNA expression [5]. ADAM17 is involved in the processing of several cell surface molecules [1] and membrane-bound TNFα [9,10]. A dysregulated shedding activity of ADAMs is an important factor in a number of pathologies, such as inflammation, neurodegenerative and cardiovascular diseases, asthma, and cancer [1,3,8,11,12,13]. Our group recently demonstrated that ADAM10 and ADAM17 are involved in ectodomain shedding of the cell adhesion molecule L1CAM in retinoblastoma (RB) cell lines, potentially influencing L1CAM-mediated effects on RB tumor formation and migration capacity in vivo, as well as apoptosis and cell proliferation in vitro [14].

Retinoblastoma is the most common primary malignant intraocular tumor in children [15,16,17]. The human retina includes six different types of neurons and Müller glia cells, originating from multipotent retinal progenitor cells [18,19,20]. The cell-of-origin of retinoblastoma could be identified as derived from a cone precursor cell [21,22,23,24,25]. Without treatment, RB tumor cells may disseminate beyond the eye and develop metastatic spread through the optic nerve into the central nervous system or lead to systemic metastases [18]. Drug delivery routes such as intra-arterial, intravitreal, or intracameral injections significantly increased eye preservation rates and reduced systemic chemotherapy, but enucleation was still the final intervention when all other therapies failed [26,27]. Tumor treatment is often limited due to massive side effects of the therapeutics and the development of resistant tumor cells leading to relapses or the development of secondary cancers [28]. Therefore, developing new and adjacent therapy strategies for the optimization of RB management is a major challenge. In this context, ADAMs already represent important drug targets for the treatment of different human diseases. Different molecules, e.g., monoclonal antibodies, have been developed to inhibit ADAM proteins [29], rendering ADAM10 and ADAM17 potentially interesting targets in RB tumor therapy. 

In the study presented, we analyzed the expression of ADAM10 and ADAM7 and their regulating miRNAs in RB cell lines and patient tumors and set out to unravel the effects of a lentiviral ADAM10 and ADAM17 single and double knockdown on RB cell viability, proliferation, apoptosis, anchorage-independent growth and AKT phosphorylation in vitro as well as tumor formation capacity, invasiveness, and dissemination in vivo. 

## 2. Results 

### 2.1. ADAM10 and ADAM17 Are Differentially Expressed in Retinoblastoma

**Figure 1:**
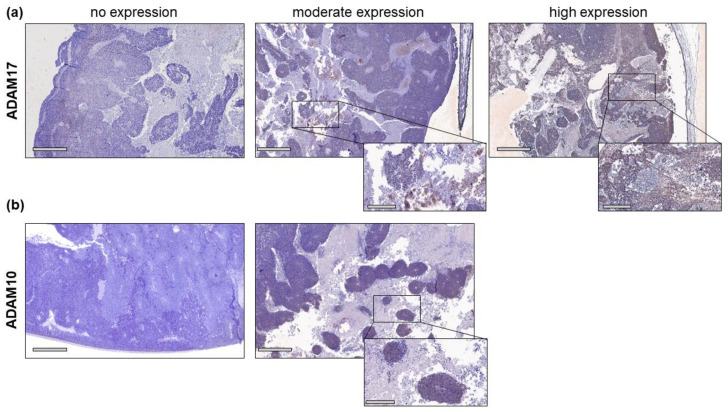
Histological analysis of primary RB tumors. ADAM17 (a) and ADAM10 (b) expression in exemplary paraffin sections of human RB patient tumors. Immunohistochemistry was detected by diaminobenzidine (brown signal) and hematoxylin counterstaining (blue nuclei staining). Scale bars, 600 µM and 200 µM (magnified insets).

Immunohistochemical stains of RB patient tumor sections revealed a moderate (13 out of 20, 65%) to high expression (4 out of 20, 20%) of ADAM17 in most of the tumors analyzed (Figure 1a), while three out of 20 (15%) tumors displayed negative for ADAM17. By contrast, no high ADAM10 expression could be identified, and a moderate expression was only detected in eight out of 20 (40%) tumors analyzed (Figure 1b). The majority of RB tumors were negative for ADAM10 (12 out of 20, 60%).

Table 1 summarizes the clinical and pathological characteristics of 20 RB patients analyzed. The cohort included 11 (55%) males and 9 (45%) females. In order to correlate the clinical and pathological parameters with ADAM10 and ADAM17 expression, RB tumors were categorized into non-expressing, weakly expressing, expressing, and highly expressing ADAM10 and ADAM17 specimens. 

No statistically significant differences could be detected between ADAM10 expression and clinical pathological characteristics of RB tumors. For ADAM17, however, statistical analyses confirmed a significant correlation between ADAM17 expression levels and laterality (*p* = 0.038) as well as International Classification for RB (ICRB) staging (*p* = 0.042) (Table 1). ADAM17 expression was significantly higher in bilateral compared to unilateral RB tumors, whereby all unilateral RBs were non-hereditary cases. According to the International Classification for RB, most eyes (n = 14) analyzed fell into ICRB group E (70%) with extensive retinoblastoma occupying >50% of the globe or invasion of the postlaminar optic nerve, five eyes were classified as group D (25%), and one eye as group B (5%). Statistical analysis revealed that ADAM17 expression was significantly higher in stage E tumors compared to stage D tumors. No statistically significant differences of ADAM17 expression in relation to sex, age at diagnosis, invasiveness, largest tumor base and treatment were discernible (Table 1). Further details of the correlation between ADAM17 expression and the different clinical parameters are summarized in Table 1.

### 2.2. ADAM10 and ADAM17 Single and ADAM10/17 Double Knockdown Influences Cell Viability, Proliferation and Growth of Y79 and WERI-Rb1 Retinoblastoma Cell Lines

**Figure 2:**
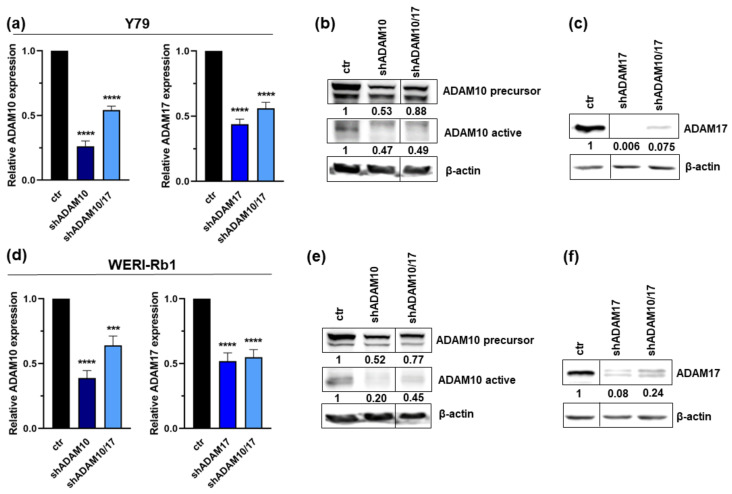
Verification of ADAM10 and ADAM17 knockdown efficiency. Efficient stable, lentiviral ADAM10 (shADAM10), ADAM17 (shADAM17) and ADAM10/17 (shADAM10/17) double knockdown was verified by quantitative real-time PCR (**a**,**d**) and western blot analyses in Y79 (**b**,**c**) and WERI-Rb1 cells (**e**,**f**). Indicated intensity ratios relative to ß-actin, used as a loading control, were calculated using MICRO MANAGER 1.4 software. Values are means of three independent experiments ± SEM. *** *p* < 0.001 and **** *p* < 0.0001 statistical differences compared to the control (ctr) group calculated by Student’s *t*-test.

We performed lentiviral ADAM10 and ADAM17 single and double knockdown experiments in the RB cell lines Y79 and WERI-Rb1, both exhibiting highest endogenous ADAM levels [14]. Testing different ADAM10 and ADAM17 specific shRNA clones separately or in combination, we achieved efficient ADAM knockdown levels as confirmed by quantitative real-time PCR (Figure 2a,d) and western blot analysis (Figure 2b,c,e,f). 

**Figure 3:**
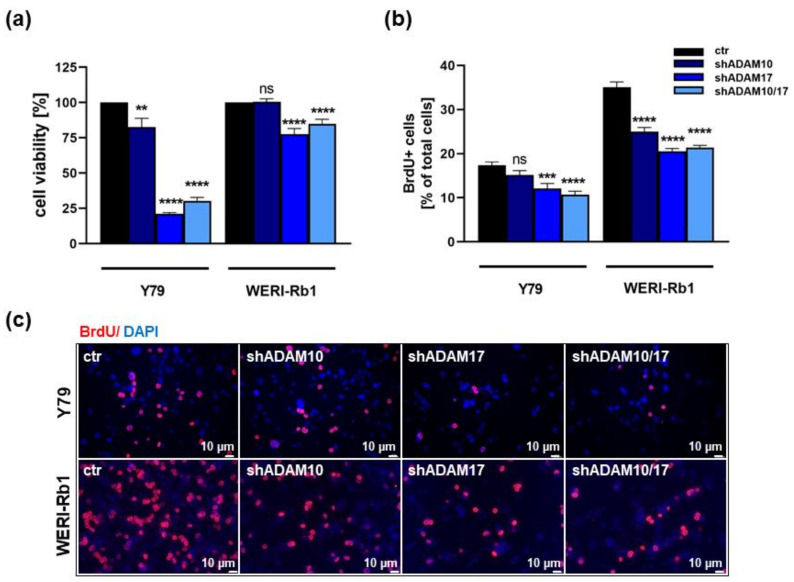
Effects of ADAM10/17 single and double knockdown on cell viability and proliferation of RB cells. Stable ADAM10 (shADAM10), ADAM17 (shADAM17) and ADAM10/17 (shADAM10/17) knockdown decreases cell viability and proliferation levels of Y79 and WERI-Rb1 cells compared to control cells (ctr) as revealed by WST-1 assays (**a**), and BrdU stains (**b**,**c**). Values are means of three independent experiments ± SEM. ns *p* > 0.05; ** *p* < 0.01; *** *p* < 0.001 and **** *p* < 0.0001 statistical differences compared to the control (ctr) group calculated by Student’s *t*-test.

As revealed by WST-1 assays and BrdU cell counts, both RB cell lines investigated exhibited significantly lower cell viabilities (Figure 3a) and decreased proliferation rates (Figure 3b,c) following ADAM10 and ADAM17 single as well as ADAM10/17 double knockdown. The viability of WERI-Rb1 cells and proliferation rates of Y79 cells remained, however, unaffected after ADAM10 knockdown. ADAM10/17 double knockdown did not increase single knockdown effects on cell viability and proliferation.

**Figure 4:**
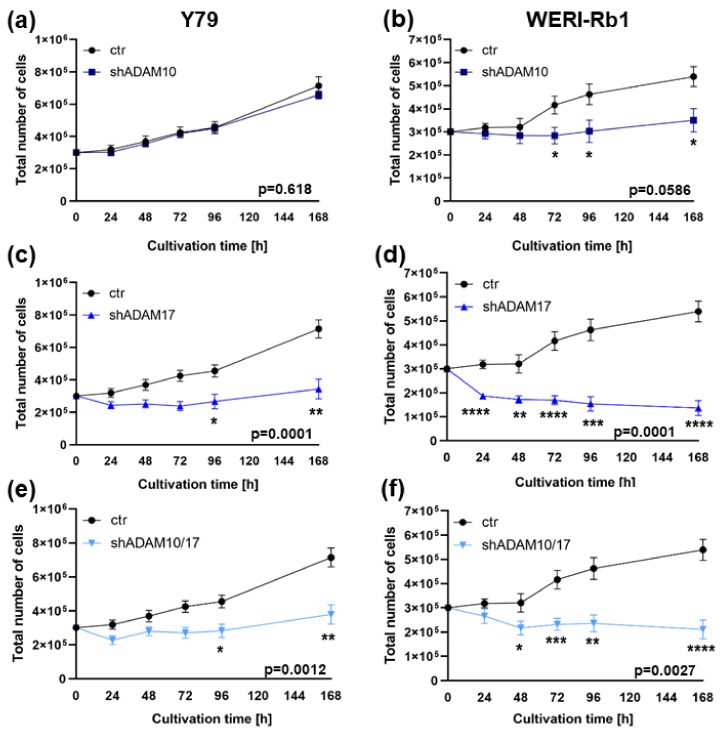
Effects of ADAM10/17 single and double knockdown on cell growth of RB cell lines. Stable ADAM10 (shADAM10), ADAM17 (shADAM17) and ADAM10/17 (shADAM10/17) knockdown decreases growth of Y79 (**a**,**c**,**e**) and WERI-Rb1 cells (**b**,**d**,**f**) compared to control cells (ctr) as revealed by growth curve analysis. Values are means of three independent experiments ± SEM. * *p* < 0.05; ** *p* < 0.01; *** *p* < 0.001 and **** *p* < 0.0001 statistical differences compared to the control (ctr) group calculated by Student’s *t*-test.

In both RB cell lines investigated, additional growth curve analyses (Figure 4a–f) after ADAM10 and ADAM17 single or ADAM10/17 double knockdown mainly confirmed the effects on cell proliferation shown above. Changes in overall cell growth did not reach significance after ADAM10 single knockdown (Figure 4a,b) being, however, highly significant after ADAM17 knockdown (*p* = 0.0001; Figure 4c,d) and to a lesser extend also following ADAM10/17 double knockdown (*p* = 0.0012 and *p* = 0.0027; Figure 4e,f). In accordance with the BrdU results, changes in overall cell growth after ADAM10 knockdown could only be shown in WERI-Rb1 cells, whereas ADAM17 knockdown induced significant effects in both RB cell lines investigated.

### 2.3. ADAM10 and ADAM17 Single and ADAM10/17 Double Knockdown Induces Caspase Dependent Apoptosis and Reduces Anchorage Independent Growth of RB Cell Lines

**Figure 5:**
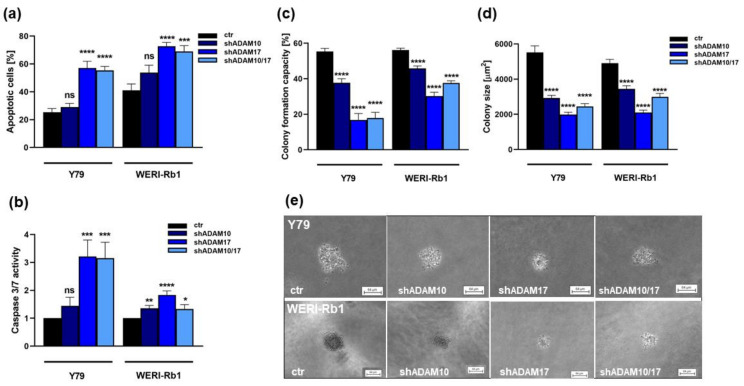
Effects of ADAM10/17 single and double knockdown on apoptosis and anchorage independent growth of RB cell lines. Stable ADAM10 (shADAM10), ADAM17 (shADAM17) and ADAM10/17 (shADAM10/17) knockdown increases caspase mediated apoptosis levels of Y79 and WERI-Rb1 cells compared to control cells (ctr) as revealed by DAPI cell counts (**a**) and caspase3/7 assays (**b**). Both RB cell lines show significantly reduced colony formation capacity (**c**) and colony sizes (**d**,**e**) after ADAM10 and ADAM 17 single or double knockdown as revealed by soft agarose assays. Values are means of three independent experiments ± SEM. ns > 0.05; * *p* < 0.05; ** *p* < 0.01; *** *p* < 0.001 and **** *p* < 0.0001 statistical differences compared to the control (ctr) group calculated by Student’s *t*-test.

ADAM17 single and ADAM10/17 double knockdown resulted in a significant increase in apoptosis levels of both RB cell lines investigated, while the number of apoptotic cells did not significantly change after ADAM10 knockdown (Figure 5a). Additional caspase assays revealed that ADAM-induced apoptosis is significantly caspase-3/7 mediated in both RB cell lines investigated (Figure 5b). ADAM10 knockdown, however, did not significantly increase caspase-3/7 activity in Y79 cells (Figure 5b). 

Compared to their parental counterparts, ADAM-depleted Y79 and WERI-Rb1 cells showed a significantly reduced colony formation capacity, displaying significantly smaller colonies in soft agarose assays testing for changes in anchorage-independent growth capability (Figure 5c,d). 

### 2.4. ADAM17 Single and ADAM10/17 Double Knockdown Decreases Tumorigenicity and Migration Potential of RB Cells In Vivo

**Figure 6:**
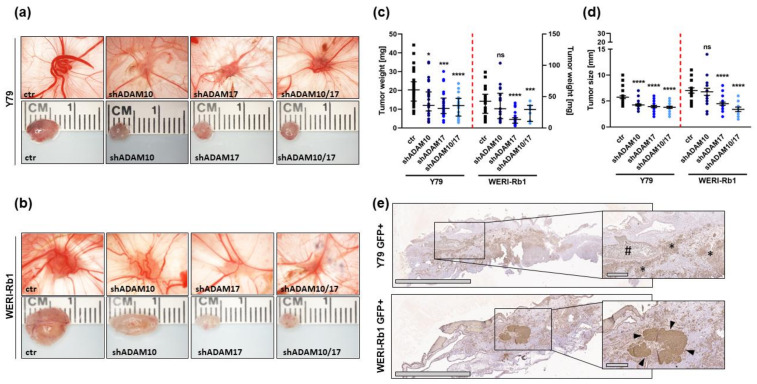
Effect of lentiviral ADAM10/17 single and double knockdown on tumor formation of RB cell lines in vivo as revealed by chick chorioallantoic membrane (CAM) assays. Photographs of CAM tumors in situ (upper row) and ruler measurements (in cm) of excised CAM tumors (lower row) 7 days after inoculating the Y79 (**a**) and WERI-Rb1 (**b**) RB cell onto the CAM. Quantification of weight (**c**) and size (**d**) of CAM tumors developing from ADAM10 (shADAM10), ADAM17 (shADAM17) and ADAM10/17 (shADAM10/17) depleted Y79 and WERI-Rb1 cell lines in comparison the control cells (ctr). Values are means of at least three independent experiments ± SEM. ns *p* > 0.05; * *p* < 0.05; *** *p* < 0.001; and **** *p* < 0.0001 statistical differences compared to the control group calculated by Student’s *t*-test. (**e**) GFP antibody staining of stably GFP expressing Y79 and WERI-Rb1 cells in CAM tumor paraffin sections. Immunohistochemistry was revealed using diaminobenzidine (brown signal) and hematoxylin counterstaining (blue nuclei staining). Scale bars, 2 mm and 300 µM (magnified insets). #: blood vessel in CAM mesoderm, *: GFP-positive (GFP+) RB tumor cells, arrowheads: solid GFP-positive RB tumor mass.

To investigate whether ADAM knockdown in vitro effects on cell growth influence RB cells’ tumor growth and migration potential in vivo, we used the chicken in ovo chorioallantoic membrane (CAM) assay as a model system. ADAM10 or ADAM17 single or ADAM10/17 double depleted Y79 or WERI-Rb1 cells and control cells were inoculated onto the CAM of EDD10 chicken embryos. Photo-documentation of CAM tumors developing from inoculated RB cells (Figure 6a,b) and quantification of tumor weight (Figure 6c) and size (Figure 6d) revealed that ADAM17 single and ADAM10/17 double depleted RB cells develop significantly smaller tumors (Figure 6a,b) than control cells, with lower weight and size (Figure 6c,d). By contrast, CAM tumors developing from ADAM10-depleted RB cells did not display significant changes in weight or size at all (WERI-Rb1 cells) or only to a lesser extent (Y79 cells). No significant changes in tumor formation capacity were discernible. Thus, ADAM knockdown effects on RB tumor formation in vivo confirmed the in vitro effects on cell growth, displaying high effects after ADAM17 and ADAM10/17 and only moderate after ADAM10 knockdown. The human RB origin of the CAM tumors was verified immunohistochemically. GFP antibody stains against inoculated, GFP-labeled RB cells revealed invaded tumor cells within the mesoderm of the CAM tissue (Figure 6e).

**Figure 7:**
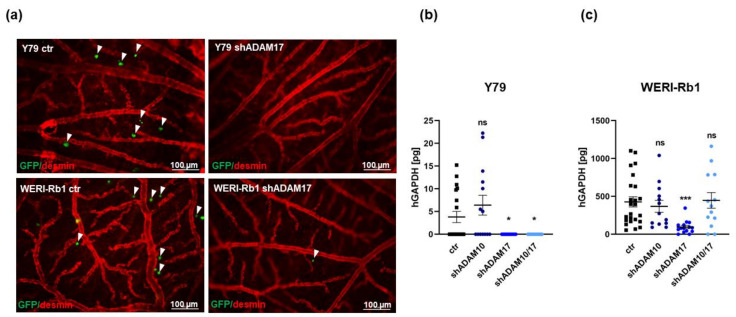
Effects of stable, lentiviral ADAM10/17 single or double knockdown on RB cell migration in vivo. (**a**) Desmin stains of blood vessels (red) in representative CAM whole mounts showing extravasated GFP-labeled Y79 and WERI-Rb1 cells (green, marked with arrowheads) after ADAM17 knockdown (shADAM17) in comparison to control cells (ctr), scale bars: 100 μm. Real-time quantification of human GAPDH (hGAPDH) content, normalized against 18S RNA, in lower CAM punches 5 days after intravenous injection of ADAM10 (shADAM10), ADAM17 (shADAM17) and ADAM10/17 (shADAM10/17) depleted Y79 (**b**) and WERI-Rb1 (**c**) human RB cell lines and control cells (ctr). Values are means of at least three independent experiments ± SEM. ns *p* > 0.05; * *p* < 0.05 and *** *p* < 0.001 statistical differences compared to the control group calculated by Student’s *t*-test.

To test for changes in migratory potential following ADAM knockdown, GFP-labeled Y79, and WERI-Rb1 cells were injected into a CAM vein. ADAM17 depleted RB cells showed clearly reduced extravasation from the CAM vasculature into the surrounding tissue (Figure 7a) and displayed a significantly lower migration rate compared to their respective controls as revealed by human GAPDH real-time PCR analyses of lower CAM punches (Figure 7b,c). In both cell lines, no significant change in migration capacity was discernible after ADAM10 depletion (Figure 7b,c), and a significant migratory effect of an ADAM10/17 double knockdown could only be detected in Y79 cells (Figure 7b). These results indicate that ADAM17 but not ADAM10 depletion alone is sufficient to decrease tumorigenicity and migration potential of RB cells in vivo.

### 2.5. ADAM10/17 Single and Double Knockdown Decreases L1CAM Shedding in RB Cell Lines

**Figure 8:**
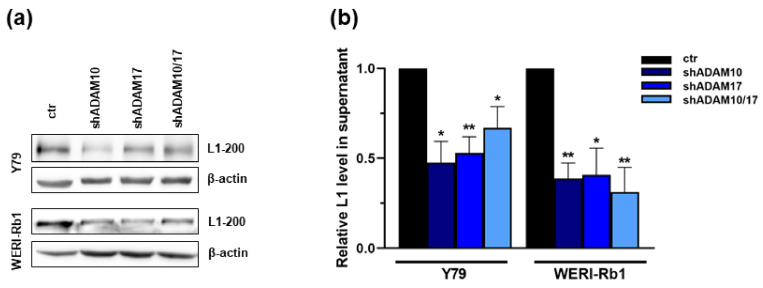
Analysis of L1CAM ectodomain shedding after ADAM10/17 single and double knockdown in Y79 and WERI-Rb1 cell lines. Western blot analysis of ADAM mediated L1CAM ectodomain (L1–200) shedding in cell culture supernatant of Y79 and WERI-Rb1 cells 72 h after ADAM10 (shADAM10), ADAM17 (shADAM17) single and ADAM10/17 (shADAM10/17) double knockdown in comparison to control cells (ctr). Representative western blots of L1 shedding upon ADAM knockdown (**a**) and quantification of the L1CAM ectodomain expression reveals a significant reduction of L1-200 shedding after ADAM10/17 single and double knockdown in the RB cell lines Y79 and WERI-Rb1 (**b**). Indicated intensity ratios relative to ß-actin, used as a loading control, were calculated using MICRO MANAGER 1.4 software. Values are means of three independent experiments ± SEM. * *p* < 0.05 and ** *p* < 0.01 statistical differences compared to the control (ctr) group calculated by Student’s *t*-test.

The soluble ectodomain of L1CAM, a known ADAM target, is believed to stimulate cell survival, protect cells from apoptosis, and mediate cell migration [30,31,32,33], effects likewise effected by ADAM10 and ADAM17 knockdown. A previous study by our group revealed that L1CAM depletion in RB cells reduces cell viability and cell growth and concomitantly induces apoptosis [14]. As in other cancer cells, ectodomain shedding of L1CAM is mediated by ADAM10 and ADAM17 [34,35], we investigated the expression of the soluble L1CAM ectodomain in cell culture supernatant after ADAM10/17 single and double knockdown. Expression levels of the soluble L1CAM ectodomain were significantly reduced in WERI-Rb1 and Y79 RB cell culture supernatant after ADAM10/17 single and double knockdown, with a median reduction of up to 50% (Figure 8a,b). These data show that also in RB cell lines, the soluble L1CAM ectodomain is released by ADAM10 and ADAM17 shedding, and thus, L1CAM could be one of the downstream effectors after ADAM10 or ADAM17 knockdown.

### 2.6. ADAM17 Knockdown Influences AKT Signaling

**Figure 9:**
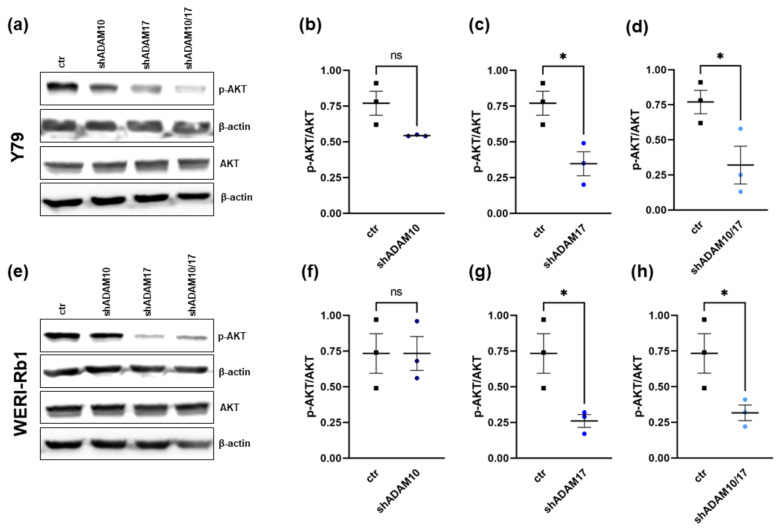
AKT and phospho-AKT (pAKT) expression levels after shRNA-mediated ADAM10/17 single (shADAM10 and shADAM17) or double knockdown (shADAM10/17) in the RB cell lines Y79 and WERI-Rb1 as revealed by western blot analysis. Representative western blots showing AKT and p-AKT expression levels (**a**,**e**) and quantification of the p-AKT/AKT ratio (**b**–**d**,**f**–**h**) after ADAM10/17 single and double knockdown in the RB cell lines Y79 and WERI-Rb1. Indicated intensity ratios relative to ß-actin, used as a loading control, were calculated using MICRO MANAGER 1.4 software. Values are means of three independent experiments ± SEM. ns *p* > 0.05 and * *p* < 0.05 statistical differences compared to the control group calculated by paired Student’s *t*-test.

The serine/threonine kinase AKT plays a key role in the PI3K signaling pathway, thereby modulating many downstream proteins involved in the survival, proliferation, migration, metabolism, and angiogenesis processes of tumor cells [36]. Recently, it has been shown that liver-specific ADAM10/17 double-deficient mice exhibit decreased AKT phosphorylation [37]. Therefore, we investigated AKT and p-AKT expression after ADAM10/17 single and double knockdown in order to reveal the AKT pathway as a possible ADAM signaling mechanism in RB cells. Phospho-AKT levels were indeed significantly decreased following ADAM17 knockdown in both RB cell lines investigated (Figure 9a,c,e,g), whereas ADAM10 knockdown did not influence the AKT phosphorylation status of the cells (Figure 9b,f). ADAM10/17 double knockdown also led to a significant reduction of AKT phosphorylation, most likely based on ADAM17 depletion (Figure 9d,h). Total AKT expression remained unaffected by all ADAM KD conditions (Figure 9a,e). Taken together, the induced in vitro and in vivo effects following ADAM17 and ADAM10/17 knockdown seem to be mediated by a lack of AKT phosphorylation and activation, respectively, and in turn, triggered regulation of downstream pathways.

### 2.7. MiRNAs Involved in the Regulation of ADAM10 and ADAM17 Expression

**Figure 10:**
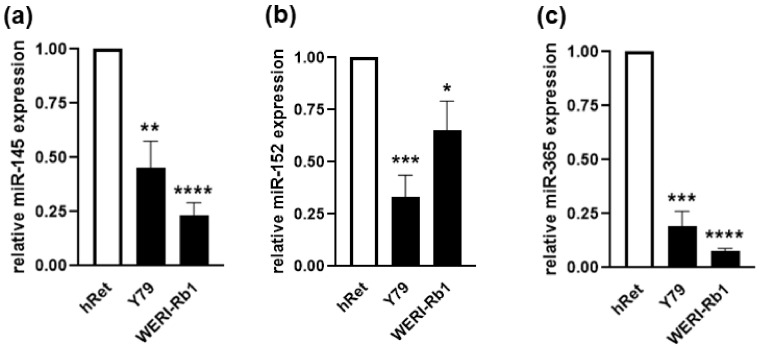
MiRNA-145, miRNA-152 and miRNA-365 expression levels in parental WERI-Rb1 and Y79 cell lines as revealed by real-time PCR (**a**–**c**). Values are means of at least three independent experiments ± SEM. * *p* < 0.05; ** *p* < 0.01; *** *p* < 0.001; and **** *p* < 0.0001 statistical differences compared to the control group calculated by Student’s *t*-test.

Attempting to decipher the mechanisms regulating the expression of ADAMs in RB, expression patterns of known ADAM10 or ADAM17 regulating miRNAs (miRs) were analyzed in WERI-Rb1 and Y79 RB cell lines. Compared to the healthy human retina, all three miRs investigated were significantly downregulated in both RB cell lines (Figure 10a–c), displaying concordantly upregulated ADAM expression levels as reported previously by our group [14].

**Figure 11:**
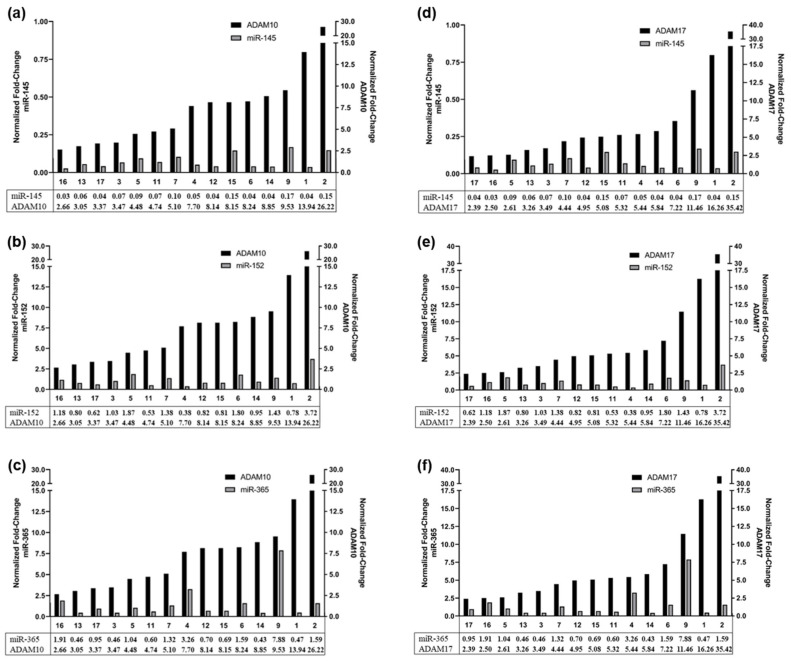
Correlation of miRNA-145, miRNA-152 and miRNA-365 expression with ADAM10 (**a**–**c**) and ADAM17 (**d**–**f**) expression levels in 15 individual RB patients as revealed by real-time PCR.

In order to reveal if the expression of these miRs also negatively correlates with ADAM10 and ADAM17 expression patterns in primary RBs, 15 RB patients’ tumor samples were analyzed. Real-time PCR analysis revealed upregulated ADAM10 (Figure 11a–c) and ADAM17 (Figure 11d–f) expression with simultaneously reduced miR-145 expression levels (Figure 11a,d) for all 15 RB patients. Expression levels of miR-152 (Figure 11b,e) and miR-365 (Figure 11c,f) were patient dependent and partially correlated with ADAM expression. These correlation patterns suggest that at least miR-145 is a potential regulator of ADAM10 and ADAM17 in retinoblastoma.

## 3. Discussion

A disintegrin and metalloproteases (ADAMS) comprise a Zn^2+^-dependent superfamily of secreted and membrane-bound sheddases, acting as “molecular scissors” as they proteolytically cleave the extracellular domain (ectodomain) of cell surface proteins, a process referred to as ectodomain shedding [29,38,39]. Ectodomain shedding controls the availability of the soluble active form of functional proteins such as growth factors, cytokines, cytokine receptors, adhesion proteins, and signaling molecules ([40]; for review: see [41,42]). Thus, ADAMs play a pivotal role in numerous physiological and pathological processes, including cell proliferation, tumorigenesis, and tumor metastasis, and have been suggested as promising drug targets in the search for new alternative therapies in many fields of medicine (for review: [29,43]). The important role of ADAMs—revealed by pre- and perinatal lethality of the respective knockout mice [44,45,46,47]—not only comprises control of mammalian development in terms of heart development, angiogenesis and neurogenesis, but also cell-cell communication, signaling, inflammation, cell adhesion and migration [40,48].

Physiologically, ADAM10 and ADAM17 are expressed in a large variety of tissues comprising, e.g., heart, prostate, kidney, and small intestine, as well as fetal lung, liver, and brain [49]. Aberrant expression or dysregulation of ADAMs has been linked to inflammatory and cardiovascular diseases as well as neurodegenerative disorders and numerous cancer entities [29,50]. A previous study by our group revealed significantly increased ADAM10 and ADAM17 protein and RNA expression levels in retinoblastoma cell lines, as well as upregulated RNA levels in RB patient tumor samples as compared to the healthy human retina [14]. Ebsen et al. likewise reported comparatively high expression levels of ADAM10 in Jurkat, pancreatic adenocarcinoma, Hela, fibrosarcoma, and Hodgkin’s lymphoma cell tumor cell lines [49]. Along this line, upregulated expression of ADAM17 and/or overexpression of ADAM10 has been associated with tumor progression as well as poor prognosis of numerous human cancers, e.g., gastric, colon, breast, pancreatic and lung cancer and hepatocellular carcinoma (for review see [39,41,43]). In the study presented, we approached the expression of both ADAMs immunohistochemically and revealed a moderate to high ADAM17 and a distinctly weaker ADAM10 protein expression in RB patients’ tumor sections. Moreover, ADAM17 expression significantly correlated with the bilaterality of RB tumors and high ICRB stages, whereas ADAM10 showed no correlation with clinical parameters. Fitting our data, ADAM10 and ADAM17 (over) expression levels likewise significantly correlates with tumor grade in glioma, and both ADAMs are discussed as prognostic markers and therapeutic targets [51,52,53].

In the study presented, WERI-Rb1 and Y79 RB cell lines displayed significantly lower cell viability and decreased proliferation rates following shRNA-mediated ADAM10 and ADAM17 single as well as ADAM10/17 double knockdown (KD). Moreover, ADAM17 single KD and ADAM10/17 double KD, but not ADAM10 KD alone, significantly increased apoptosis levels. Our results are consistent with previous studies showing that ADAM17 silencing significantly decreased the growth of nasopharyngeal cancer cells [54] as well as cell viability, growth, and proliferation rate of breast cancer cells [55] and suppressed cell viability of esophageal squamous cell carcinoma [56,57]. Furthermore, ADAM17 overexpression likewise increased proliferation and significantly decreased apoptosis levels of melanoma cells [58]. Moreover, silencing of ADAM10 reduced cell proliferation in HepG2 hepatocellular carcinoma cells, ligamentum flavum cells, and nasopharyngeal cancer cells [59,60,61,62]. In contrast to our results, silencing of ADAM10 alone has been shown to be sufficient to increase apoptosis in hepatocellular carcinoma cells [61,63].

Our study presented revealed that increased apoptosis after ADAM17 single and ADAM10/17 double knockdown is caspase-3/7 mediated. Consistently, overexpression of ADAM17 in melanoma and hepatocellular carcinoma cells resulted in reduced apoptosis accompanied by a significant decrease in caspase-3 cleavage [58,64]. Moreover, ADAM17 KD in vascular endothelial cells has been shown to offer protection from retinal ischemia-reperfusion (IR) injury of ADAM17 Cre-flox mice by decreasing apoptotic cell death and active caspase-3 levels of vascular endothelial cells leading to decreased vascular degeneration [65]. Thus, ADAM17 KD during IR injury can be beneficial for preventing the consequences of ischemia in retinal tissue, which is in line with the positive influence on RB tumorigenicity seen after ADAM17 KD in retinoblastoma. In contrast to our data, revealing that ADAM10 KD alone does not significantly increase caspase-3/7 activity in RB cells, ADAM10 depletion has been shown to increase caspase-3 cleavage of hepatocellular carcinoma cells [63]. Thus, the effect of ADAM silencing and the relevance of individual ADAM family members for carcinoma cell growth and survival seems to be cell-type-specific.

Testing for changes in anchorage-independent growth, soft agarose assays revealed that ADAM10 and ADAM17 depleted Y79, and WERI-Rb1 retinoblastoma cells displayed a significantly reduced colony formation capacity. These findings are in good accordance with a study by Liu et al. (2017) reporting on significantly inhibited colony formation in hepatocellular carcinoma cells following ADAM10 depletion [59].

In our study, ADAM17 single and ADAM10/17 double, but not ADAM10 depleted RB cells developed significantly smaller tumors in vivo than control cells. Moreover, ADAM17-depleted RB cells displayed a significantly lower migration rate compared to their respective controls. No significant change in migration capacity was discernible after ADAM10 depletion, indicating that ADAM17 but not ADAM10 depletion alone is sufficient to decrease tumorigenicity and migration potential of RB cells in vivo.

Furthermore, we noticed differences in blood vessel density around the CAM tumors developing upon inoculation of control or ADAM-depleted RB cells. CAM tumors developing from ADAM10 and ADAM17 depleted RB cells seemed to have reduced blood vessel density compared to control cells. In accordance with our observations, it could be shown that retinal blood vessels in ADAM17 hypomorphic mice require ADAM17 activity in angiogenesis [66], whereas ADAM10 inhibition induced vascular sprouting and density in vivo. However, further analyses regarding the involvement of ADAM10 and ADAM17 in blood vessel sprouting in RB tumor development are needed to address the question properly.

Consistent with our data, shRNA silencing of ADAM17 significantly inhibited the invasion and migration of breast and nasopharyngeal cancer cells [54,55]. Similarly, inhibition of ADAM17 with an inhibitory antibody likewise suppressed motility in human pancreatic cancer cells in vitro and also significantly delayed tumorigenesis in a murine model [67], and ADAM17 silencing significantly suppressed hypoxia-induced migration of keratinocytes [68]. Further along this line, exosomal ADAM17 was shown to promote the migration of colorectal cancer cells [69]. Moreover, in accordance with our result, ADAM17 overexpression in exosomes increased the hepatic metastases rates of colorectal cancer cells in a murine model, whereas ADAM17 downregulation in exosomes eliminated the liver metastasis stimulation ability of exosomes on colorectal cancer cells [69]. In contrast to our study, ADAM10 single KD was sufficient to inhibit nasopharyngeal cancer migration [60], and treatment with a monoclonal antibody against ADAM10 inhibited lymphoma growth in a xenograft model and gastrointestinal tumor growth in a genetic mouse model [70]. Moreover, siRNA silencing of ADAM10 significantly suppressed cell migration and invasion of hepatocellular carcinoma cells in wound healing and transwell invasion assays in vitro, as well as tumor growth in vivo in a murine xenograph model [59,61]. Further along this line, overexpression of ADAM10 augmented cell migration, whereas a proteolytically non-active form of ADAM10 blocked cell migration of HEK cells [30]. Moreover, ADAM10 was correlated with glioma growth and invasiveness [52]. Thus, the significance of ADAM10 and ADAM17 for tumor growth, tumor cell migration, invasion, and metastasis seems to be cell type dependent, and our data suggest that ADAM17 plays a pivotal role in retinoblastoma development.

Previous studies reported that ADAM10 and ADAM17 are involved in the ectodomain shedding of the cell adhesion molecule L1CAM [34,35]. In the study presented, expression levels of the soluble L1CAM ectodomain were reduced by up to 50% in the cell culture supernatant of ADAM10/17 single- and double-depleted WERI-Rb1 and Y79 cells. These findings indicate that in RB cell lines, the soluble L1CAM ectodomain is likewise released by ADAM10 and ADAM17 shedding, confirming previous data by our group [14].

In our study, ADAM17 KD significantly decreased AKT phosphorylation. A resulting attenuation of AKT signaling might be one of the potential mechanisms by which ADAM17 KD mediates downstream effects, such as reduced proliferation and increased caspase-dependent apoptosis in RB cells. Fittingly, in ADAM17-silenced breast cancer cells, p-AKT levels are likewise significantly decreased [55], and forced expression of ADAM17 enhanced the phosphorylation of AKT in hepatocellular carcinoma cells without affecting the expression of total AKT [64]. Moreover, ADAM17 KD decreases AKT signaling in glioblastoma cells [71]. Our experiments revealed that ADAM10 silencing did not influence the AKT phosphorylation status of RB cells. Similarly, liver-specific ADAM10 deficient mice likewise exhibited no changes in AKT phosphorylation status [37]. By contrast, expression levels of p-AKT were significantly decreased in ligamentum flavum cells following ADAM10 silencing [62], and silencing or ectopic expression of ADAM10 resulted in a marked increase in the phosphorylation of Akt in hepatocellular carcinoma cells [61,63].

Analyzing the potential regulation of ADAM expression in RB cell lines and patient tumors by miRNAs (miRs), in the study presented, miR-152, a miR discussed to target ADAM17 and regulate its expression in other cancer entities [51,72], was differentially expressed in the RB cell lines and in a subset of RB tumor samples investigated. In RB tumors, the correlation of miR-152 levels with ADAM10 and ADAM17 expression was patient-dependent, but miR-152 expression correlated with ADAM expression in RB cell lines displaying significantly decreased miR-152 and concomitantly upregulated ADAM17 and ADAM10 levels. Corresponding to our data, expression patterns of miR-152 and ADAM17 were opposite in HUVEC cells [73] and expression levels likewise inversely correlated in non-small cell lung cancer tissues [72]. While reinvestigating the expression pattern of miR-145, another miR that targets ADAM17 [74], we found its levels to be significantly decreased in WERI-Rb1 and Y79 RB cells and patient tumors, confirming the findings of a former study, reporting on downregulated miR-145 levels in RB cell lines and tissues [75]. The authors, however, identified ADAM19 as a direct target of miR-145, whereas our expression studies suggest an additional regulation of ADAM17 and ADAM10 by this miR in RB cells, as ADAM17 and ADAM10 levels were concomitantly upregulated in the RB cells investigated. In this context, miR-145 was described as a direct target of ADAM17 in glioblastoma cells, and reciprocal regulation of ADAM17 and miR-145 is believed to drive the invasiveness of this tumor entity [71]. Moreover, we demonstrated significantly downregulated expression levels of miR-365, known to target ADAM10 [59,76], in WERI-Rb1 and Y79 RB cells as compared to the healthy human retina. In these RB cell lines, ADAM10 and ADAM17 levels are significantly upregulated [14], suggesting the regulation of both ADAMs by this miR. The correlation of miR-365 with ADAM10 and ADAM17 expression in RB tumor samples appeared to be patient-dependent, but both ADAMs seem to be targeted by miR-365, at least in a subset of RB tumors. Inverted expression patterns indicate that both ADAMs, which share 30% amino acid identity in humans, might be targets of the respective miRs in RB cells as already described for other tumor entities. This hypothesis is strengthened by a study describing a reciprocal negative feedback loop between miR-145 and ADAM17 expression in renal cell carcinoma, in which miR-145 negatively regulates ADAM17 and ADAM17, in turn, negatively regulates miR-145 expression [74]. Moreover, it has been shown that the knockdown of ADAM10 or ADAM17 mimics the effects induced by overexpression of their respective regulating miRs [54,59,74,77]. In nasopharyngeal carcinoma cells, miR-145 expression was likewise significantly downregulated, whereas ADAM17 protein expression was upregulated, and ADAM17 had been verified as a target of miR-145 [54]. Furthermore, ADAM17 is a target of miR-145 in glioma cells [77]. Similarly, miR-365 expression is low, whereas ADAM10 expression is upregulated in triple-negative breast cancer cells compared to normal breast cancer cells, and ADAM10 has been identified as a direct target of miR-365 in these [76] as well as in colorectal cancer and hepatocellular carcinoma cells [59,78]. Moreover, high ADAM10 levels inversely correlated with miR-365 levels were reported as being significantly downregulated in hepatocellular carcinoma cell lines and tumor tissues [59]. Taken together, our data suggest that at least ADAM17, being upregulated in RB cells and tissue, might be a promising target for future retinoblastoma therapy strategies as well as a potential diagnostic biomarker.

The therapeutic potential of ADAM10 and ADAM17 has already been exploited [3,79,80], and targeting these ADAMs genetically by antibodies or pharmacological inhibitors has been shown to suppress cell proliferation and tumor growth in various pre-clinical cancer models ([81]; for review, see [39,41,79,82]). Although these inhibitors displayed promising effects, they often did not reach beyond phase three in clinical trials due to side effects either resulting from non-specificity or side effects such as musculoskeletal and liver toxicities or deep vein thrombosis [41]. Ongoing clinical tests with new inhibitors will show if these side effects are attributable to the non-specificity leading to off-target effects or to the fact that ADAM10/17 cleaves a broad spectrum of substrates involved in numerous essential biological processes.

ADAM17 has already been discussed as a valuable diagnostic and prognostic biomarker for colorectal cancer metastasis [68,83], early-stage ovarian cancer [84], and malignant CNS tumors [85]. It has been shown that the ectodomain of ADAM17 can shed itself as a consequence of a cleavage mediated by ADAM8, and soluble ADAM17 was demonstrated to be still active [80]. ADAM17 was detected in a culture supernatant of cancer cell lines and primary tumor cultures as well as blood serum of cancer patients [83,84,86]. Elevated levels of ADAM17 were observed in the cerebrospinal fluid of patients with neoplastic meningitis, mild cognitive impairments (MCI), and Alzheimer’s disease (AD) [87,88,89]). Thus, we set out to determine if ADAM17 might be detectable in supernatants of RB cell lines, primary RB cell cultures, and RB patient liquid biopsies such as aqueous humor and blood. Western blot and ELISA analyses revealed that ADAM17 expression levels are below the detection limit in RB cell culture supernatants and patients’ aqueous humor samples. By contrast, ADAM17 was detectable in blood samples; however, no significant differences in ADAM17 protein levels were discernible comparing RB patients and control blood samples. Fitting our results, ADAM17 plasma protein levels did not significantly differ between MCI and AD patient and control samples, whereas ADAM17 plasma activity was significantly higher in patients [88]. Together, these data further strengthen the role of ADAM17 as a potential cancer biomarker.

## 4. Material and Methods

### 4.1. Human Retina and Retinoblastoma Samples

Patient retinoblastoma (RB) samples and post-mortem healthy human retinae were used for comparative expression studies. The study also includes a retrospective case series of 20 eyes from individual children diagnosed with intraocular retinoblastoma. Diagnosis was confirmed pathologically after enucleation. The data collected included patient’s age at diagnosis, gender, laterality, TNM staging, International Classification of Retinoblastoma (ICRB) classification [90], tumor treatment, largest tumor base, and *RB1* mutation status.

### 4.2. Pathological Characteristics and Definitions

In this study, tumors were divided into four groups according to the age at the time of diagnosis: (1) diagnosis under 1 year of age (<1), (2) diagnosis between 1 and 2 years of age (<2), (3) diagnosis between 2 and 3 years of age (<3), and (4) diagnosis after 3 years of age (>3).

The classification of tumor treatment was divided into two groups: (1) untreated tumors (defined as primary enucleation without any prior treatment) and (2) treated tumors (defined as secondary enucleation after systemic or local chemotherapy with/or without thermal therapy, cryotherapy, brachytherapy, and external beam radiation therapy). The staging for this study was performed in line with the ICRB staging system and the AJCC/UICC staging system (TNM) [91,92].

### 4.3. Cell Lines and Culture

The human RB cell lines Y79 [93] and WERI-Rb1 [94], originally purchased from the Leibniz Institute DSMZ (German Collection of Microorganisms and Cell Cultures), were kindly provided by Dr. H. Stephan. The cell lines were cultivated as suspension cultures in Dulbecco’s modified Eagle’s medium (DMEM; PAN-Biotech, Aidenbach, Germany) with 15% fetal bovine serum (FBS; PAN-Biotech), 100 U penicillin/mL and 100 µg streptomycin/mL (Invitrogen, Darmstadt, Germany), 4 mM L-glutamine (Gibco, Karlsruhe, Germany), 50 µM β-mercaptoethanol (Carl Roth, Karlsruhe, Germany) and 10 µg insulin/mL (PAN-Biotech, Aidenbach, Germany) at 37 °C, 10% CO_2_ and 95% humidity. Human embryonic kidney cells (HEK293T) were grown as adherent cell culture in DMEM (PAN-Biotech, Aidenbach, Germany) with 10% FBS (PAN-Biotech, Aidenbach, Germany), 4 mM L-glutamine (Gibco, Karlsruhe, Germany), 100 U penicillin/mL, and 100 µg streptomycin/mL (Gibco, Karlsruhe, Germany) at 37 °C, 5% CO_2_ and 95% humidity. No approval from an ethics committee was required for work with the human cell lines.

### 4.4. Generation of Lentiviral Particles and Stable ADAM10 and ADAM17 Single and Double Knockdown

In order to generate lentiviral particles, 6 × 10^6^ human embryonic kidney cells (HEK293T) were transfected with 6 µg of each of the following plasmid DNAs: (I) packaging vectors pczVSV-G [95] and pCD NL-BH [95], (II) shADAM10 *clone*TRCN0000418144 for transduction of both RB cell lines, (III) shADAM17 cloneTRCN0000052171 for transduction of WERI-Rb1 cells, (IV) shADAM17 cloneTRCN0000052172 for transduction of Y79 cells (for knockdown experiments “Mission shRNA Plasmid DNA” with a pLKO.1puro backbone was used from Sigma-Aldrich), (V) pPRIME-CMV-Neo-FF3 (p234) as a negative control for all knockdown experiments [96] or (VI) GFP expression vector (pCL7EGwo, provided by Dr. H. Hanenberg) each in the presence of 45 µg polyethyleneimine (PEI, branched, Sigma-Aldrich, St. Louis, Missouri, USA) in DMEM medium. After 24 h the medium was changed to Iscove’s Modified Dulbecco’s medium (IMDM, Pan-Biotech, Aidenbach, Germany) with 10% FBS and 1% penicillin/streptomycin and 72 h after transfection viral supernatants were harvested, filtered (0.45 µm filter) and cryopreserved.

For stable transduction, 1.25 × 10^6^ RB cells were seeded in DMEM medium. After 24 h the medium was removed, and the cells were transfected with either only ADAM10 or ADAM17 virus particles, control virus particles, or a combination of ADAM10 and ADAM17 virus particles (half of each virus titer) for the double knockdown approach, each in the presence of polybrene (5 µL per ml lentivirus; H9268, Sigma-Aldrich, München, Germany). After 24 h, DMEM medium with supplements (twice the volume of the virus particles) was added, and 48 h later, the medium was completely changed, and the cells were incubated for another 72 h.

### 4.5. RNA Extraction and Quantitative Real-Time PCR

RNA isolations from RB cells and CAM tissue were performed using the NucleoSpin^®^ RNA II Kit (Macherey & Nagel, Düren, Germany) and the miRNeasy Kit (Qiagen, Hilden, Germany), respectively.

For quantitative real-time PCR analyses, cDNA was synthesized with the QuantiTect Reverse Transcription Kit (Qiagen, Hilden, Germany) according to the manufacturer´s protocol. For analysis of ADAM10 and ADAM17 expression, an SYBR^TM^ green PCR assay (Applied Biosystem, Darmstadt, Germany) was used with specific primers 5′-CACGAGAAGCTGTGATTGCC-3′ (forward) and 5′-TCCGGAGAAGTCTGTGGTCT-3′ (reverse) for *ADAM10*, 5′-AGGATGCTTGGGATGTGAAGA-3′ (forward) and 5′-GTGAAAAGGTGTGCCAAGCA-3′ (reverse) for *ADAM17* as well as 5′-ACCCACTCCTCCACCTTTGA-3′ (forward) and 5′-CTGTTGCTGTAGCCAAATTCGT-3′ (reverse) for human *GAPDH* (h*GAPDH*) as an endogenous control.

Real-time PCR reactions were conducted in triplicates in 20 µL of SYBR^TM^ green PCR Mastermix (Applied Biosystem, Darmstadt, Germany) performing 40 cycles of the following program: 95 °C for 15 min; 94 °C for 15 s, 55 °C for 30 s and 70 °C for 34 s.

For micro-RNA expression analyses, a miScript PCR Starter Kit (# 2181193; Qiagen, Hilden, Germany) was used, following the manufacturer´s protocol. The designated miScript HiSpec Buffer (Qiagen, Hilden, Germany) for quantification of mature miRNA was used along with specific primers for hsa-miR-145-5p (GUCCAGTTTTCCCAGGAATCCCT), hsa-miR-152-5p (AGGTTCTGTGATACACTCCGACT), hsa-miR-365a-5p (AGGGACTTTTGGGGGCAGATGTG) and *5.8S* RNA (5′-CTACGCCTGTCT GAGCGTCGCTT-3′) as an endogenous control. The reactions were performed in duplicates using a QuantStudio^TM^ 3 real-time PCR system (ThermoFisher, Darmstadt, Germany), with the following program: 95 °C for 15 min; 94 °C for 15 s, 55 °C for 30 s, and 70 °C for 34 s and 40 cycles. RNA isolation from chicken chorioallantoic membrane (CAM) tissue punches (see below) was performed as described previously [43]. Quantitative real-time PCR analyses were performed and quantified following the protocol published previously [43]. The following human Taqman Gene Expression Assays (Applied Biosystems) were used: GAPDH (Hs99999905_m1) and 18S (Hs99999901_s1). The latter was used as an endogenous control for human and chicken samples.

### 4.6. Western Blotting

For western blot analyses, cells were washed in phosphate buffer saline (PBS, PAN-Biotech GmbH, Aidenbach, Germany) and lysed in radioimmunoprecipitation assay (RIPA) buffer plus supplements [97] for 30 min at 4 °C on a shaker and centrifuged at 10,000 rpm at 4 °C for 30 min. Afterward, protein concentrations were measured by bicinchoninic acid assay (BCA; Thermo-Scientific, Oberhausen, Germany) according to the manufacturer’s protocol. Equal amounts of protein extracts were separated on a 10% SDS-PAGE and transferred onto nitrocellulose membranes. Membranes were incubated with primary antibodies against ADAM10 (1:1000; #14194; Cell Signaling, Danvers, USA), ADAM17 (1:500; PA5-17079; Invitrogen, Darmstadt, Germany), L1-ectodomain (1:1000; L4543; Sigma-Aldrich, München, Germany), AKT (1:1000; #4685; Cell Signaling Technology, Danvers, USA), p-AKT (1:500; #9271; Cell Signaling Technology, Danvers, USA) and β-actin (1:1000; #4967; Cell Signaling Technology, Danvers, USA) at 4 °C overnight. HRP-conjugated species-specific secondary antibodies (goat-anti-rabbit; P0448 and rabbit-anti-mouse; P0260; DAKO, Glostrup, Denmark) were used in dilutions of 1:10,000 at room temperature for 1 h on a tabletop shaker. The signals were developed by adding Western Bright Chemiluminescence Reagent (Cytiva, Buckinghamshire, UK).

### 4.7. Cell Viability Assays

In order to determine cell viability, 4 × 10^4^ cells in 100 µL medium were seeded in a 96-well plate in two triplicates. After 72 h of incubation, 10 µL of a water-soluble tetrazolium (WST-1) salt solution (Sigma-Aldrich, München, Germany) was added to each well, and cells were incubated at 37 °C for a designated period. Quantification of the formazan dye produced by viable cells was performed by measuring the absorbance at 450 nm in a microplate reader.

### 4.8. Growth Kinetic

In order to determine growth kinetics in a 24-well plate format, 3 × 10^5^ cells were seeded in 500 µL DMEM medium (PAN-Biotech, Aidenbach, Germany) with supplements in triplicates, and the number of vital cells was counted manually every 24 h (6 time points: 0 h, 24 h, 48 h, 72 h, 96 h, and 168 h) in a Neubauer chamber using the trypan blue exclusion method.

### 4.9. Cell Proliferation and Apoptosis Detection

Cell proliferation was determined by 5-Bromo-2’-deoxyuridine (BrdU; Sigma, Hamburg, Germany) incorporation. For BrdU immunochemistry, 4 h prior to PFA fixation 5 µM BrdU was added to the cells. Afterward, cells were permeabilized and incubated with a rat anti-BrdU antibody (1:1000; ab6326; Abcam, Cambridge, UK), and the BrdU signal was visualized using a goat anti-rat secondary antibody labeled with Alexa Fluor 594 (1:1000; Molecular Probes, Eugene, USA).

For each experiment, five coverslips were stained, and the percentages of proliferating cells were calculated by setting BrdU-positive cells in relation to the total amount of DAPI-positive cells.

### 4.10. Caspase-Glo 3/7 Assay

In order to analyze the caspase 3 and 7 cleavage activity after ADAM knockdown, a caspase-Glo 3/7 assay (Promega, Madison, WI USA) was used. Therefore, 1.5 × 10^5^ ADAM10 or/and ADAM17 were depleted, and control cells were seeded in 500 µL growth medium supplemented with 2% FBS in a 24-well plate format and incubated overnight. After 24 h, 60 µL of each cell suspension was mixed with 60 µL of caspase-3/7 reagent and seeded in a white 96-well plate for 2 h at room temperature protected from light. Afterward, luminescence was measured with an Orion II microplate luminometer (Berthold Detection Systems, Pforzheim, Germany) following the manufacturer´s instructions. Measurements were performed three times in five replicates.

### 4.11. Colony Formation and Soft Agarose Assay

For soft agarose assays, 5 × 10^4^ RB cells were suspended in 2 mL DMEM/F12 medium (Sigma-Aldrich, München, Germany) supplemented with 10% fetal calf serum, 100 U penicillin/mL and 100 µg streptomycin/mL, 4 mM L-glutamine, 50 µM β-mercaptoethanol, 10 µg insulin/mL and 0.7% agarose (Roth, Karlsruhe, Germany). This cell suspension was layered on 2 mL 1% agarose, containing DMEM/F12 medium with the same supplements as indicated above, and cultured in 6-well plates at 37 °C, 10% CO_2,_ and 95% humidity were maintained over a period of 3 weeks. Colony formation, starting from single cells seeded in the upper layer of soft agarose, was quantified after 3 weeks of incubation, and assays were repeated three times. The colony formation capacity (%) for each cell line was calculated by counting the number of colonies and viable single cells in six visual fields at a 10× magnification in triplicates. Colony size was measured by capturing images using a Nikon Eclipse TS2 microscope equipped with a digital camera and IC Measure 1.0 software (Nikon, Düsseldorf, Germany). Six colonies per well were measured for the determination of colony size.

### 4.12. CAM Assays

In order to study the effects of ADAM knockdowns on tumor formation and migration capacity in vivo, ADAM-depleted RB cells and control cells were inoculated on the extraembryonic chorioallantoic membrane (CAM) of chick embryos on embryonic developmental day (EDD)10 mainly following the protocols published by Zijlstra and Palmer [98,99]. Ten eggs per group were inoculated with 1 × 10^6^ cells suspended in 50 µL PBS in at least three independent experiments. Seven days after grafting, at EDD17, grown tumors were excised, measured, weighted, and photographed as described previously [100].

Intravenous injection of GFP-labeled Y79 and WERI-Rb1 control and ADAM knockdown cells was carried out on EDD12, as described previously by our group [100]. Fifty microliters of cell suspension (5 × 10^5^ cells in DMEM medium without any supplements) were injected into the allantoic vein of each embryo. Injections were performed under a dissection microscope in order to monitor if the injected cells were carried by the blood flow. Five days after injection (EDD12-17), the chicken embryos were sacrificed, and six punches of the ventral CAM, opposing the injection site, were collected and processed as described previously [99,101]. Successful migration after injection was monitored by the identification of the GFP-labelled cells via fluorescence microscopy of the CAM punches. If at least two punches contained detectable GFP-labelled cells, all 6 CAM punches per egg were pooled and processed for further analyses. RNA isolations and quantification of hGAPDH of the pooled CAM punch tissue were performed as described previously [100].

For wholemount immunofluorescent staining of CAM vessels, CAM punches were fixed in 4% PFA (Sigma-Aldrich, München, Germany) overnight at 4 °C on a tabletop shaker in 24 well plates. CAM punches were washed three times with Tris-buffered saline (TBS; 150 mM NaCl; 20 mM Tris-HCl; Carl-Roth, Karlsruhe, Germany) containing 0.1% Triton X-100 (Sigma-Aldrich, München, Germany) for 30 min at room temperature on a tabletop shaker. Blocking was performed by incubation with PBS containing 3% bovine serum albumin (BSA; Carl-Roth, Karlsruhe, Germany) for 1 h at room temperature with gentle shaking. The first antibody detecting chicken desmin (D33;ab8470, Abcam, Cambridge, UK) was diluted 1:20 in PBS with 3% BSA and incubated in a humidified chamber at 4 °C overnight. CAM punches treated with blocking buffer (PBS + 3% BSA) only served as a negative control. The next day, tissue samples were washed in PBS with 1% Triton X-100 for 10 min and three times with PBS with 1% Triton X-100 and 20% fetal bovine serum (FBS, PAN-Biotech, Aidenbach, Germany) for 1 h each on a tabletop shaker at room temperature. Alexa-fluor^®^594 goat anti-mouse IgG (Molecular Probes, Eugene, USA) was diluted 1:1000 in 300 µL of PBS, and CAM punches were incubated in a 24-well plate format on a shaker overnight at 4 °C. The following day, samples were washed three times with PBS containing 1% Triton X-100 at room temperature for 30 min each on a tabletop shaker. Finally, the CAM punches were carefully placed on slides and mounted with DACO fluorescent mounting media (DAKO, Glostrup, Denmark) under 10 mm glass plates. Subsequent fluorescence microscopy was carried out with a Nikon ECLIPSE E600 microscope and NIS Elements Imaging 5.20.02 software (Nikon, Düsseldorf, Germany).

### 4.13. Immunohistochemistry

For ADAM10 and ADAM17 immunohistochemical localization in formalin-fixed, paraffin-embedded retinoblastoma and to immunohistochemically localize GFP labeled cells in fixed and embedded CAM tumors, sample sections were deparaffinized and steamed for 1 h in citrate buffer (pH 6 for ADAM17) or EDTA buffer (pH 9 for ADAM10 and GFP) to improve antigen retrieval. Sections were preincubated for 5 min with 3% H_2_O_2_ in methanol to quench endogenous peroxidase activity, rinsed 3 times in PBS, and incubated for 20 min at room temperature with normal blocking serum provided in the Vectastain Elite ABC kit (Biozol, Eching, Germany). Immunostaining was performed using a rabbit monoclonal antibody against ADAM10 (#14194; Cell Signaling Technology, Danvers, USA), ADAM17 (PA5-17079; Invitrogen, Darmstadt, Germany), and GFP (2955S; Cell Signaling Technology, Danvers, USA) at a dilution of 1:100 (ADAM10), 1:100 (ADAM17) and 1:200 (GFP) in antibody dilution buffer provided in the Vectastain kit (Biozol, Eching, Germany) at 4 °C overnight in a humidified chamber. After 3 rinses in PBS, sections were incubated with the biotinylated secondary antibody provided in the Vectastain kit (Biozol, Eching, Germany) for 30 min at room temperature, washed in PBS, and incubated for 30 min with the Vectastain Elite ABC reagent (Biozol, Eching, Germany). The reaction was visualized by 3,3´-diaminobenzidine (DAB; Sigma-Aldrich, München, Germany) staining, and the sections were counterstained with hematoxylin. As controls, in all cases, antibody dilution buffer was substituted for the primary antisera to test for nonspecific labeling. No specific cellular staining was observed when the primary antiserum was omitted. Images were acquired by a slide scanner (Leica, Wetzlar, Germany) and viewed using the Aperio Image Scope Software (Leica).

The stained slides were peered by eyeballing, taking into account different staining intensities (I) defined as null (0), mild (1), moderate (2), or strong (3) expressed, and the percentage (P) of tumor with ADAM10 or ADAM17 positive cells. The quick score (QS) was then calculated as I × P (from 0 to 300). Not expressed was defined as QS 0, weakly expressed as QS 1-20, expressed as QS 21-60, and highly expressed as QS > 60.

### 4.14. Statistical Analysis

All assays were performed at least in triplicates. Statistical analyses were performed using GraphPad Prism 9. Data represent means ± SEM of three independent experiments from independent RB cell cultures. Results were analyzed by a Student’s *t*-test and considered significantly different if *p*-value < 0.05 (*), *p*-value < 0.01 (**), *p*-value < 0.001 (***) or *p*-value < 0.0001 (****). Statistics on the growth curves was performed using a free web interface http://bioinf.wehi.edu.au/software/compareCurves/, accessed on 1 January 2022 which uses the “compare growth curves” function from a statistical modeling package called statmod, available from the “R Project for Statistical Computing”: http://www.r-project.org, accessed on 1 January 2022 previously described elsewhere [102]. Statistical correlation analyses (Kruskal–Wallis rank sum *p*-values) of clinical parameters with ADAM10 and ADAM17 expression were performed in the R statistical environment, version 3.2.0.16.

## Figures and Tables

**Table 1:** Clinical and pathological characteristics of RB patients’ tumors stratified by ADAM17 expression in immunohistochemically stains.

	Highly Expressed *n* (%)	Expressed *n* (%)	Weakly Expressed *n* (%)	Not Expressed *n* (%)	*N*	*p*-Value *
**Patients**	4 (20)	7 (35)	6 (30)	3 (15)	20	
**Sex**						0.45
female	2 (50)	4 (57)	2 (33)	1 (33)	9	
male	2 (50)	3 (43)	4 (67)	2 (67)	11	
**Laterality**						**0.038**
unilateral	2 (50)	5 (71)	6 (100)	3 (100)	16	
bilateral	2 (50)	2 (29)	0 (0)	0 (0)	4	
**Age at diagnosis**						0.48
<1 year	1 (25)	2 (29)	0 (0)	0 (0)	3	
<2 years	0 (0)	4 (57)	2 (33)	1 (33)	7	
<3 years	3 (75)	1 (14)	3 (50)	2 (67)	9	
>3 years	0 (0)	0 (0)	1 (17)	0 (0)	1	
**ICRB**						**0.042**
C	1 (25)	0 (0)	0 (0)	0 (0)	1	
D	0 (0)	0 (0)	4 (67)	1 (33)	5	
E	3 (75)	7 (100)	2 (33)	2 (67)	14	
**Invasiveness**						0.52
none	2 (50)	2 (29)	3 (50)	2 (67)	9	
invasion	2 (50)	5 (71)	3 (50)	1 (33)	11	
**Largest tumor base**						0.69
<15mm	2 (50)	3 (43)	5 (83)	1 (33)	11	
>15mm	2 (50)	4 (57)	1 (17)	2 (67)	9	
**Treatment**						0.59
none	3 (75)	7 (100)	5 (83)	3 (100)	18	
chemo	1 (25)	0 (0)	1 (17)	0 (0)	2	

n: number in each group, N: total number, ICRB group C: discrete local disease with minimal subretinal or vitreous seeding, ICRB group D: diffuse disease with significant vitreous or subretinal seeding, ICRB group E: extensive retinoblastoma. * Kruskal–Wallis rank sum *p*-value.
